# Risk factors for hepatitis B transmission in South Brazil

**DOI:** 10.1590/0074-02760170043

**Published:** 2017-08

**Authors:** Vagner Reinaldo Zingalli Bueno Pereira, Jonas Michel Wolf, Camila Albani da Silva Luz, Gláucia Zuleide Stumm, Thais da Rocha Boeira, Josiane Galvan, Daniel Simon, Vagner Ricardo Lunge

**Affiliations:** 1 Programa de Pós-Graduação em Biologia Celular e Molecular Aplicada à Saúde, Universidade Luterana do Brasil, Canoas, RS, Brasil; 2 Laboratório de Diagnóstico Molecular, Universidade Luterana do Brasil, Canoas, RS, Brasil; 3 Universidade de Caxias do Sul, Caxias do Sul, RS, Brasil; 4 Prefeitura Municipal de Caxias do Sul, Serviço Municipal de Infectologia, Caxias do Sul, RS, Brasil

**Keywords:** chronic hepatitis B, South Brazil, epidemiologic factors

## Abstract

**BACKGROUND:**

Hepatitis B virus (HBV) infection is a major public health problem in Brazil. Several risk factors are involved in HBV infection and their identification by a rational and essential approach is required to prevent the transmission of this infection in Brazil.

**OBJECTIVES:**

To evaluate risk factors associated with HBV infection in South Brazil.

**METHODS:**

A total of 260 patients with HBV and 260 controls from Caxias do Sul (state of Rio Grande do Sul, Brazil) participated in this study. All participants were given a standard questionnaire to yield the sociodemographic information and to identify HBV risk factors. HBV infection was detected by HBsAg test in all participants.

**FINDINGS:**

HBV infection in these cases was strongly associated with history of a family member HBV-infected, mainly mother [odds ratio (OR) = 4.86; 95% confidence intervals (CI): 1.69–13.91], father (OR = 5.28; 95% CI: 1.58–17.71), and/or siblings (OR = 22.16; 95% CI: 9.39–52.25); sharing personal objects (OR = 1.40; 95% CI: 1.37–2.38); and having history of blood transfusion (OR = 2.05; 95% CI: 1.10–2.84).

**CONCLUSIONS:**

HBV infection was strongly associated with having a family member infected with hepatitis B, sharing personal objects, and having history of blood transfusion.

Hepatitis B virus (HBV) infection is one of the most important human diseases and 240–280 million people are infected with chronic HBV worldwide (WHO 2015, 2016). Moreover, patients infected with HBV can develop cirrhosis, hepatocellular carcinoma, and other hepatic dysfunctions. More than 600,000 people die every year due to clinical hepatic complications after the initial HBV infection (Niederau 2014).

HBV is highly prevalent in Asia, but is also frequent in populations from Sub-Saharan Africa and Oceania islands. In contrast, North America, Australia, and Western Europe have very low endemicity. In South America, endemicity ranges from high to low depending on the country (Roman et al. 2014). Brazil has moderate to low endemicity depending on the region, state, and city. In general, HBV prevalence increases from the South to the North (Souto 2016). The northern states — Acre, Rondônia, Pará, Amazônia, and Mato Grosso — have the highest endemicity in the country. In contrast, the southernmost regions have a low HBV prevalence, but the states of Santa Catarina and Paraná were reported to have moderate to high endemicity (Souto 2016). Previous studies have also demonstrated that some specific populations (Italian descendants) contribute to high hepatitis B rates in some regions and cities of South Brazil (Bertolini et al. 2012, Gusatti et al. 2015).

HBV has been controlled by immunisation worldwide for more than 20 years. In Brazil, the vaccination initiated in the 1990s starting with the children and was gradually extended to the remaining population. It is estimated that 50% of the population has received HBV vaccine and effective immunisation occurred in approximately 60% of the vaccinated people (Souto 2016, Ximenes et al. 2015). Consequently, HBV is still being largely spread and recently infected people have been observed in all age groups. Besides these new infections, several patients are infected with progressive chronic HBV since a long time. The results of the massive serological screening have revealed anti-HBc and HBsAg as markers for HBV infection and 12.7% and 0.7% of the total population from Brazil, respectively, was positive for these markers (Souto 2016).

Despite the low prevalence of HBV and availability of vaccination programs, the South Brazilian Region presented the highest HBV prevalence rates in the country (17.2 cases per 100,000 inhabitants). HBV frequency also increases with age in this geographic region — 1.6% anti-HBc positive people were aged between 10 and 19 years *versus* 11.3% anti-HBc positive people were aged between 20 and 69 years (Ximenes et al. 2015). In the city of Caxias do Sul [state of Rio Grande do Sul (RS)], an even higher HBsAg seroprevalence (1.6%) was observed in a large sample population (Menegol & Spilki 2014).

HBV is transmitted by contact with infected blood and body fluids. Several studies have already reported the transmission by sharing infected objects. Therefore, drug users, who share syringes and other objects contaminated with blood, usually have a high risk of HBV infection (Degenhardt et al. 2016, Matos et al. 2013). This situation also occurs in case of patients or health professionals infected by handling HBV-contaminated medical devices (Bârlean et al. 2014, Diercke et al. 2015). On the contrary, transmission within the family is less reported (Shepard et al. 2006), although some previous studies have demonstrated that sharing personal objects with family members (e.g., safety razor, dishes, cutlery, glasses, face towels, and toothbrush) is strongly associated with HBV transmission (Lobato et al. 2006, Nazzal & Sobuh 2014). The present case-control study aimed to investigate the association between epidemiological risk factors and chronic HBV in a city (Caxias do Sul) with high HBV prevalence in South Brazil.

## MATERIALS AND METHODS

*Sampling* – The sample size was estimated as previously described (Lwanga & Lemeshow 1991). The statistical power of 80%, 5% significant level (two-tailed test), and 15% frequency of exposure in the case group, was considered to give an expected odds ratio (OR) of 2.5 at 95% confidence interval (CI). To the selected samples (208 cases and 208 controls), 25% more samples were added for including the potential confounders, amounting to 260 cases and 260 controls that were matched for age, sex, and self-defined ethnicity. The case group was composed of patients with HBV routinely treated in the Specialised Centre for Health in the city of Caxias do Sul, from September 2014 to March 2016. The patients were from the urban and rural areas of this city and visited the medical service for periodic examinations and/or treatment for chronic hepatitis B (all patients were HBsAg positive and anti-HBc IgG positive). Controls were obtained by convenience sampling at a local University Hospital (General Hospital) during the same period. All control subjects were HBsAg negative (confirmed by the lateral flow immunochromatographic test, VIKIA^®^, Rio de Janeiro, RJ, Brazil, HBsAg Kit). In addition, control participants with the previous history of some infectious and chronic diseases, such as primary sclerosing cholangitis, Wilson’s disease, hepatitis, cirrhosis, AIDS, hypertension, cancer, and autoimmune diseases, were excluded from the study, according to self-declaration and/or identification in the medical records of the hospital. All participants also signed informed consent form and the study was previously approved by the Ethical Committee of Lutheran University of Brazil with the protocol 32075314.3.0000.5349.

*Data collection* – A self-administered anonymous questionnaire with questions on sociodemographic and risk factor information was structured according to previous reports (Nazzal and Sobuh 2014, Pereira et al. 2009). All surveys were supervised by a trained interviewer. Further, levels of aspartate aminotransferase (AST) and alanine aminotransferase (ALT) were obtained from medical records from the most recent tests at the time of interview.

*HBV laboratory analysis* – Blood samples were collected from each participant in tubes containing ethylenediamine tetraacetic acid (EDTA) anticoagulant. Subsequently, they were centrifuged to separate the plasma and plasma samples were stored at -20°C. HBsAg was detected in all the samples and anti-HBc IgG and IgM were detected only in samples of patient with chronic HBV. Commercial lateral flow immunochromatographic kits were used for anti-HBc immunological assays, according to the manufacturer’s instructions (Architect^®^, Abbott Diagnostics, Sligo, Ireland).

DNA was extracted from blood samples by a method involving adsorption on silica. Real-time polymerase chain reaction assays (PCR) (for HBV detection) were carried out using a Step One Plus™ platform (Applied Biosystems^®^, Foster City, CA, USA) with previously reported cycling and temperature conditions (Pas et al. 2000). The results were interpreted as positive and negative, based on the cycle thresholds (C_t_s) from the amplifications plots. Further, viral load (VL) was determined in HBV-DNA positive samples using control samples with defined VL as described previously (Pas et al. 2000).

*Statistical analysis* – Data were analysed using SPSS software (Statistical Package for Social Sciences, v. 17.0, Chicago, IL, USA). The Student’s *t* test for independent samples was used to detect statistical differences between quantitative variables. Frequencies were calculated for all qualitative variables. Bivariate analysis was performed to assess the association between categorical variables and the outcome using the Fisher’s exact test or the chi-square test (as recommended). The multivariate analysis test was performed through binary logistic regression method variables associated with HBV infection in the bivariate analysis (p ≤ 0.05). The magnitudes of these associations were estimated as OR with 95% CI. The values of p < 0.05 in multivariate analysis were considered statistically significant.

## RESULTS

*The general profile of cases and controls* – The mean age of cases was 47.62 ± 12.22 years, with a predominance of people from the self-defined white ethnic group (n = 229, 88.1%) and male sex (n = 143, 55%). Most patients with HBV were also married (n = 203, 78.1%), having studied elementary school or less (n = 159, 61.1%), living in rural area of the city in childhood (n = 183, 71%), and having more than five siblings (n = 145, 55.8%). The average number of siblings for patients was 6.13 ± 3.54. The most common occupation was farming (n = 45, 17.3%), followed by metallurgy (n = 24, 9.2%), and mason (n = 22, 8.4%). In addition, a predominance of Italian descendants, when the participants were questioned about the ancestry of the four grandparents (n = 164, 63.1%), was observed in comparison to that of German (n = 18, 6.9%), Portuguese (n = 14, 5.4%), and African (n = 3, 1.2%) descendants as well as those with mixed ancestries (n = 56, 21.5%). Among the cases, this information was lacking only for five (1.9%) participants.

The control participants were matched to the cases in terms of age, self-defined ethnicity, and sex. Considering other sociodemographic characteristics, most of the controls were also married (n = 167, 64.2%) and having studied elementary school or less (n = 212, 81.5%), similar to that noted in the patients. However, the majority of the control participants lived in the urban area of the city in childhood (n = 148, 57%) and had less than five siblings (n = 136, 52.3%). In this group, the average number of siblings was 5.73 ± 3.68. Masonry was the main occupation (n = 31, 11.9%), followed by farming (n = 21, 8.1%) and metallurgy (n = 20, 7.7%). Interestingly, a predominance of people with mixed ancestors (n = 177, 68.1%) in comparison to the Caucasian (Italian, German, or Portuguese) ancestry (n = 39, 11.2%) was observed. In this group, 54 (20.8%) participants did not reveal the ethnic ancestry.

The comparison between the sociodemographic characteristics of the cases and that of the controls is presented in Table I. Marital status, education, place of residence, rural occupation, and number of siblings were significantly different between the groups (p < 0.05).

**TABLE I:** Sociodemographic variables in groups of cases and controls (Caxias do Sul, state of Rio Grande do Sul, Brazil, 2014–2016)

Variables	Cases (n = 260)			Controls (n = 260)		p
	
	n	%		n	%	
Age (years)						> 0.999
< 20	3	1.1		3	1.1	
20–40	70	26.9		70	26.9	
41–60	148	56.9		148	56.9	
61–80	38	14.6		38	14.6	
> 80	1	0.5		1	0.5	
Sex						> 0.999
Male	143	55		143	55	
Female	117	45		117	45	
Ethnic group						> 0.999
Not white	31	11.9		31	11.9	
White	229	88.1		229	88.1	
Marital status						< 0.001
Married	203	78.1		167	64.2	
Not married	57	21.9		93	35.8	
Level of schooling						< 0.001
Elementary school or less	159	61.1		212	81.5	
High school	101	38.9		48	18.5	
Place of residence in childhood						< 0.001
Rural area	183	71		112	43	
Urban	76	29		148	57	
Occupation						0.017
Farmer	45	17.3		21	8.1	
Mason	22	8.4		31	11.9	
Metallurgical	24	9.2		20	7.7	
Other	122	47		141	54.2	
Retired/housewife	47	18.1		47	18.1	
Number of siblings^*a*^						0.015
> 5	145	55.8		114	43.8	
≤ 5	112	43.1		136	52.3	

*a*: totals do not coincide due to lack of data from certain participants in the study.

Considering clinical characteristics, patients with HBV revealed an average AST level of 32.07 ± 25.31 U/L and ALT level of 33.40 ± 20.52 U/L. A total of 138 (53%) patients did not undergo any anti-HBV therapy, while 122 (47%) were being treated with medicines specific against HBV (interferon α, entecavir, tenofovir, lamivudine, and adefovir). HBV-DNA was tested in all patients and 82 (31.5%) samples were positive, among which 36 (29.5%) were treated and 46 (33.8%) were untreated. The VL in the treated patients was slightly higher (log_10_ = 4.69 ± 1.33 copies/mL) than that in the untreated controls (log_10_ = 3.91 ± 1.34 copies/mL).

*Epidemiological factors associated with HBV* – The differences between case and control groups were more evident in some risk factors of chronic HBV infection. Cases presented a much higher proportion of relatives with a history of HBV infection, such as HBV-infected mother (48.2% vs. 3.2%), father (36% vs. 2.4%), and one or more siblings (67% vs. 3.8%), than that presented by controls. The other three classical HBV risk factors — sharing personal objects (56% vs. 40%), having history of blood transfusion (15% vs. 6.9%), and using previously used syringe glass (65% vs. 46.2%) — were also more frequent in the cases than in the controls. However, other five HBV risk factors — body piercing, birthing method, previous sexually transmitted infection (STI), alcohol consumption, and illicit drug use — presented similar frequencies in the cases and controls. A non-significant statistical difference was noted between the two groups in a separate analysis on the method of drug use (sniffed, smoked, and injected) (data not shown). Surprisingly, tattooing was noted to be more frequent in the controls than in the cases (17.7% vs. 8.8%).

A bivariate comparative analysis between cases and controls was performed with all HBV transmission risk factors (Table II). The results showed that having a mother (OR = 28.18; 95% CI: 13.29–59.72), father (OR = 22.50; 95% CI: 9.54–53.05), and/or siblings (OR = 50.58; 95% CI: 25.55–100.12) infected with HBV; sharing personal objects (OR = 1.92; 95% CI: 1.35–2.73); having history of blood transfusion (OR = 3.28; 95% CI: 1.70–6.31); using glass syringe (OR = 2.18; 95% CI: 1.53–3.12); and having tattoo (OR = 0.45; 95% CI: 0.26–0.77) were associated with chronic HBV infection. In contrast, alcohol consumption, illicit drug use, body piercing, birthing method, and previous STI showed no significant association with HBV infection (p > 0.05).

**TABLE II:** Bivariate analysis of risk factor for hepatitis B transmission (Caxias do Sul, state of Rio Grande do Sul, Brazil, 2014–2016)

Variable	Cases (n = 260)			Controls (n = 260)		p	OR (95% CI)
	
	n	%		n	%		
History of mother infected (HBV)							
Yes	109	48.2		8	3.2	< 0.001	28.18 (13.29–59.72)
No	117	51.8		242	96.8	-	1.00
History of father infected (HBV)							
Yes	75	36		6	2.4	< 0.001	22.50 (9.54–53.05)
No	135	64		243	97.6	-	1.00
History of siblings infected (HBV)							
Yes	174	67		10	3.8	< 0.001	50.58 (25.55–100.12)
No	86	33		250	96.2	-	1.00
Alcohol consumption							
Yes	204	78.5		191	73.3	0.182	1.31 (0.87–1.97)
No	56	21.5		69	26.5	-	1.00
Illicit drugs use^*a*^							
Yes	29	11.2		30	11.5	0.890	0.96 (0.56–1.65)
No	231	88.8		230	88.5	-	1.00
Sharing of personal objects							
Yes	145	56		104	40	< 0.001	1.92 (1.35–2.73)
No	113	44		156	60	-	1.00
Blood transfusion history							
Yes	39	15		18	6.9	< 0.001	3.28 (1.70–6.31)
No	221	85		242	93.1	-	1.00
Previous use of glass syringe							
Yes	167	65		120	46.2	< 0.001	2.18 (1.53–3.12)
No	89	34.5		140	53.8	-	1.00
Body piercing							
Yes	5	1.9		4	1.5	0.737	1.25 (0.33–4.72)
No	255	98.1		256	98.5	-	1.00
Tattoo							
Yes	23	8.8		46	17.7	0.003	0.45 (0.26–0.77)
No	237	91.2		214	82.3	-	1.00
Type of birth							
Normal	239	93		241	95	0.388	0.73 (0.35–1.49)
Caesarean	19	7		14	5	-	1.00
Previous STI							
Yes	215	91.5		241	93.5	0.332	0.72 (0.36–1.41)
No	20	8.5		16	6.2	-	1.00

*a*: illicit drugs use (inhaled, sniffed, smoked, and injected); CI: confidence interval; HBV: hepatitis B virus; OR: odds ratio; STI: sexually transmitted infection.

All risk factors associated with HBV infection were compared in a multivariate analysis (Table III). Variables with statistically significant results were: having mother (OR = 4.86; 95% CI: 1.69–13.91), father (OR = 5.28; 95% CI: 1.58–17.71), and/or siblings (OR = 22.16; 95% CI: 9.39–52.25) with HBV; sharing personal objects (OR = 1.40; 95% CI: 1.37–2.38); and having a history of blood transfusion (OR = 2.05; 95% CI: 1.10–2.84).

**TABLE III:** Multivariate analysis of variables associated with chronic hepatitis B infection in the bivariate analysis in groups of cases and controls (Caxias do Sul, state of Rio Grande do Sul, Brazil, 2014–2016)

Variables	p	OR	95% CI
History of mother infected (HBV)	0.003	4.86	1.69–13.91
History of father infected (HBV)	0.007	5.28	1.58–17.71
History of siblings infected (HBV)	< 0.001	22.16	9.39–52.25
Sharing of personal objects	< 0.001	1.40	1.37–2.38
Blood transfusion history	0.023	2.05	1.10–2.84
Previous use of glass syringe	0.086	1.43	0.84–1.72
Tattoo	0.079	0.82	0.67–1.17
Marital status	0.072	1.33	0.82–1.74
Level of schooling	0.065	0.92	0.76–1.35
Place of residence in childhood	0.063	0.96	0.82–2.77
Occupation	0.080	0.98	0.90–2.95
Number of siblings	0.095	1.90	0.80–3.10

CI: confidence interval; HBV: hepatitis B virus; OR: odds ratio.

## DISCUSSION

HBV infection is one of the important health problems in Brazil. In South Brazil, recent data indicate that a prevalence rate higher than the average occurs in some cities and regions (Bertolini et al. 2012, Gusatti et al. 2015). Caxias do Sul is one of these cities, with 1.6% population positive towards HBsAg (Menegol & Spilki 2014). The whole city is located in the mountains of RS, a region colonised by European immigrants, mainly Italians. In this population, sociodemographic characteristics and risk factors were compared between cases and controls to identify epidemiological characteristics associated with HBV infection.

Although the controls were selected by matching the three criteria — age, sex, and self-defined ethnicity — and all the participants were from the same city and the surrounding regions, the cases group presented differences in the sociodemographic characteristics in comparison with that presented by the control group. First, the cases group presented higher rates of married and higher level of schooling than those presented by the control group. Further, more people in the cases group lived in rural areas in childhood, while those in the control group lived in the urban area. Brazilian Ministry of Health demonstrated that people in a rural area live in unfavourable conditions, often having limited access to health services, which could favour the acquisition of diseases (Brasil 2015). However, this is probably not the only explanation of this difference. Patients with HBV also presented families with more members and were mainly of the Italian descent, while most controls had families with fewer members and were of mixed ancestries. High frequency of HBV infection in cities and countryside regions colonised by Italians in Brazil has been previously reported (Bertolini et al. 2006, 2012). Approximately 1.4 million Italian immigrants arrived in Brazil between 1870 and 1920. A large percentage of these immigrants settled in the South Region, seeking job opportunities (IBGE 2010). Previous data indicate that immigrants who arrived in this region in the last century possibly introduced HBV in these regions (Bertolini et al. 2012, Gusatti et al. 2015).

Alcohol consumption and drug use (especially, when injected) were already described as important risk behaviours related to HBV infection (Ximenes et al. 2015). Both these risk factors were not associated with HBV transmission in the present study. Heavy alcohol consumption was previously associated with HBV infection in only one region (Southeast), but not in the other four regions (Central-West, North, Northeast, and South) in Brazil (Ximenes et al. 2015). Illicit drug use has also not been associated with HBV infection in a nationwide study in Brazil (Pereira et al. 2009). However, inhaled and sniffed drugs were associated with HBV in South Brazil in a more recent report (Ximenes et al. 2015). All methods of drug use (sniffed, smoked, and injected) were analysed separately and no significant difference was noted between cases and controls. A possible reason for this absence of association is the low percentage (11.3%) of drug users in the whole study.

Another classical risk factor, STI, does not have an association with HBV infection in this population. Our results are in disagreement with other studies, including multicentric population-based study in the Northeast, Central-West, and Federal Districts of Brazil (Pereira et al. 2009), Amazon riparians (Oliveira et al. 2011), and population-based survey in North, Southwest, and South of Brazil (Ximenes et al. 2015), that found association between the history of STI and HBV infection. Differences between the sample profiles of the present study and that in the other studies could justify these disagreements.

Body piercing and tattooing have also been classically associated with HBV infection (Jafari et al. 2012, Pereira et al. 2009, Yang et al. 2015). Both the factors were not associated with HBV in our study population as noted in logistic regression. A similar result was observed in a previous study in South Brazil (Ximenes et al. 2015). Notably, tattooing was more frequent in the controls (17.7%) than in the cases (8.8%) and was associated with HBV infection as noted in the bivariate analysis. Probably, a covariant (the predominance of people living in the city in the control group, 57%) influenced this association, since tattooing is more common in the people living in urban areas.

In contrast, sharing a glass syringe and blood transfusion were associated with HBV infection. These associations were already reported in other studies on populations from Brazil (Pereira et al. 2009, Ximenes et al. 2015). Further, sharing of personal objects within the family was also associated with HBV infection as previously demonstrated (Clemente et al. 2009, Lobato et al. 2006). Generally, sharing safety razor, dishes, cutlery, glasses, face towels, and toothbrush with an infected person is strongly associated with HBV transmission (Lobato et al. 2006, Nazzal & Sobuh 2014).

Meanwhile, the most interesting result was the strong association between HBV infection in patients and its occurrence in their family members (mother, father, and mainly siblings) that indicated a history of a similar infection. OR to acquire HBV infection in individuals increased by 4.86, 5.28, or 22.16 times in people with an HBV-infected mother, father, or siblings. These results demonstrate the importance of HBV transmission within the family, mainly in childhood, in South Brazil. HBV transmission in early childhood was already demonstrated in North Brazil (Paraná & Almeida 2005). A high frequency of HBsAg-positive cases was evident in siblings (75%) whose mother was positive for the same marker (p < 0.0001) in the Amazon Region. In addition, HBV markers in other family members were statistically higher in relatives of HBsAg-positive mothers (Lobato et al. 2006). This form of transmission has already been reported in other countries, such as Italy (Contini et al. 2012, , Stroffolini 2005), Germany (Deterding et al. 2012), and Portugal (Mota et al. 2009).

This study also has some limitations. It was not possible to ascertain the status of HBV infection or vaccination against hepatitis B in controls in the past. Instead, medical information about each control participant was revised and hepatitis or related diseases (cirrhosis, liver cancer, etc.) were not registered in the questionnaire and/or in the electronic data from the hospital. No evidence regarding the vaccination of controls was recorded. In addition, a more definitive evidence of the intrafamilial transmission would have been obtained if HBV samples were subjected to molecular genotyping and/or sequencing. Molecular epidemiology studies, with HBV sequencing of all family members, should be performed to better understand HBV transmission in this population.

However, the findings of this study suggest a pivotal role of intrafamilial HBV transmission in one of the most populated cities (Caxias do Sul) in South Brazil. This scenario is certainly similar in other surrounding regions that present the same geographical and sociocultural characteristics (of Italian immigration). Previous reports demonstrated that most patients with HBV in this whole region are infected with genotype D, with some cities presenting this genotype in all HBV-infected individuals (Bertolini et al. 2012, Gusatti et al. 2015). Further studies are necessary to observe if this scenario is indeed a specific epidemiological characteristic of the other cities in the South Region of Brazil.

HBV infection in a patient was strongly associated with an HBV-infected family member (mother, father, and/or siblings), suggesting that intrafamilial HBV contamination plays a pivotal role in HBV transmission in this population. Further, sharing personal objects and having a history of blood transfusion were also associated with HBV infection in the present study.

## References

[B1] Bârlean L, Săveanu I, Balcoş C (2014). Dental patients’ attitudes towards infection control. Rev Med Chir Soc Med Nat Iasi.

[B2] Bertolini DA, Gomes-Gouvêa MS, Carvalho-Mello IM, Saraceni CP, Sitnik R, Grazziotin FG (2012). Hepatitis B virus genotypes from European origin explains the high endemicity found in some areas from southern Brazil. Infect Genet Evol.

[B3] Bertolini DA, Pinho JR, Saraceni CP, Moreira RC, Granato CF, Carrilho FJ (2006). Prevalence of serological markers of hepatitis B virus in pregnant women from Paraná state, Brazil. Braz J Med Biol Res.

[B4] Brasil, Ministério da Saúde, Secretaria de Vigilância em Saúde (2015). Brasil. Sistema de Informações sobre Mortalidade. Óbitos de hepatite B. Brasil, grandes regiões e unidades federadas 2000–2007.

[B5] Clemente CM, Carrilho FJ, Pinho JR, Ono-Nita SK, Da Silva LC, Moreira RC (2009). A phylogenetic study of hepatitis B virus in chronically infected Brazilian patients of Western and Asian descent. J Gastroenterol.

[B6] Contini C, Badia L, Cultrera L, Grilli A, De Togni A (2012). Epidemiological, clinical and laboratory features of chronic hepatites B infection in a cohort of immigrant and italian patients from Ferrara, Italy. Ann Hepatol.

[B7] Degenhardt L, Charlson F, Stanaway JD, Larney S, Alexander LT, Hickman M (2016). Estimating the burden of disease attributable to injecting drug use as a risk factor for HIV, hepatitis C and hepatitis B: results from the Global Burden of Disease GBD 2013 study. Lancet Infect Dis.

[B8] Deterding K, Heidelberger S, Wiebner B, Meining K, Cornberg M, P Manns M (2012). Knowledge and management of hepatitis B virus infected patients in Germany. Dtsch Med Wochenschr.

[B9] Diercke M, Monazahian M, Petermann H, Gerlich WH, Schüttler CG, Wend U (2015). Hepatitis B outbreak in a nursing home associated with reusable lancet devices for blood glucose monitoring, Northern Germany 2010. J Med Virol.

[B10] Gusatti CS, Costi C, Halon ML, Grandi T, Medeiros AF, Silva CM (2015). Hepatitis B virus genotype d isolates circulating in Chapecó, southern Brazil, originate from Italy. PLoS ONE.

[B11] IBGE – Instituto Brasileiro de Geografia e Estatística (2010). Censo demográfico.

[B12] Jafari S, Buxton JA, Afshar K, Copes R, Baharlou S (2012). Tattooing and risk of hepatitis B: a systematic review and meta-analysis. Can J Public Health.

[B13] Lobato C, Tavares J, Rios-Leite M, Trepo C, Vitvitski L, Parvaz P (2006). Intrafamilial prevalence of hepatitis B virus in western Brazilian Amazon region: epidemiologic and biomolecular study. J Gastroenterol Hepatol.

[B14] Lwanga SK, Lemeshow S (1991). Sample size determination in health studies: a practical manual.

[B15] Matos MA, Ferreira RC, Rodrigues FP, Marinho TA, Lopes CL, Novais AC (2013). Occult hepatitis B virus infection among injecting drug users in the Central-West Region of Brazil. Mem Inst Oswaldo Cruz.

[B16] Menegol D, Spilki FR (2014). Seroprevalence of hepatitis B and C markers at the population level in the municipality of Caxias do Sul, southern Brazil. Braz J Microbiol.

[B17] Mota A, Guedes F, Areias J, Pinho L, Cardoso MF (2009). Epidemiological study of genotypes of hepatitis B virus in northern Portugal. J Med Virol.

[B18] Nazzal Z, Sobuh I (2014). Risk factors of hepatitis B transmission in northern Palestine: a case - control study. BMC Res Notes.

[B19] Niederau C (2014). Chronic Hepatitis B in 2014: Great therapeutic progress, large diagnostic deficit. World J Gastroenterol.

[B20] Oliveira CS, Silva AV, Dos Santos KN, Fecury AA, Almeida MK, Fernandes AP (2011). Hepatitis B and C virus infection among Brazilian Amazon riparians. Rev Soc Bras Med Trop.

[B21] Paraná R, Almeida D (2005). HBV epidemiology in Latin America. J Clin Virol.

[B22] Pas SD, Fries E, De Man RA, Osterhaus AD, Niesters HG (2000). Development of a quantitative real-time detection assay for hepatitis B virus DNA and comparison with two commercial assays. J Clin Microbiol.

[B23] Pereira LM, Martelli CM, Merchán-Hamann E, Montarroyos UR, Braga MC, Lima ML (2009). Population-based multicentric survey of hepatitis B infection and risk factor differences among three regions in Brazil. Am J Trop Med Hyg.

[B24] Roman S, Jose-Abrego A, Fierro NA, Escobedo-Melendez G, Ojeda-Granados C, Martinez-Lopez E (2014). Hepatitis B virus infection in Latin America: a genomic medicine approach. World J Gastroenterol.

[B25] Shepard CW, Simard EP, Finelli L, Fiore AE, Bell BP (2006). Hepatitis B virus infection: epidemiology and vaccination. Epidemiol Rev.

[B26] Souto FJ (2016). Distribution of hepatitis B infection in Brazil: the epidemiological situation at the beginning of the 21st century. Rev Soc Bras Med Trop.

[B27] Stroffolini T (2005). The changing pattern of hepatitis B virus infection over the past three decades in Italy. Dig Liver Dis.

[B28] WHO – World Health Organization (2015). Guidelines for the prevention, care and treatment of persons with chronic hepatitis B infection.

[B29] WHO – World Health Organization (2016). Hepatitis B.

[B30] Ximenes RA, Figueiredo GM, Cardoso MR, Stein AT, Moreira RC, Coral G (2015). Population-based multicentric survey of hepatitis b infection and risk factors in the North, South, and Southeast regions of Brazil, 10–20 years after the beginning of vaccination. Am J Trop Med Hyg.

[B31] Yang S, Wang D, Zhang Y, Yu C, Ren J, Xu K (2015). Transmission of hepatitis B and C virus infection through body piercing: a systematic review and meta-analysis. Medicine.

